# Epidemiological investigations of contagious caprine pleuropneumonia in selected districts of Borana zone, Southern Oromia, Ethiopia

**DOI:** 10.1007/s11250-018-1744-y

**Published:** 2018-11-05

**Authors:** Dereje Teshome, Teshale Sori, Flavio Sacchini, Barbara Wieland

**Affiliations:** 1 Yabello Pastoral and Dryland Agriculture Research Center, P.O. Box 85, Yabello, Ethiopia; 2 College of Veterinary Medicine and Agriculture, Addis Ababa University, P.O. Box 34, Bishoftu, Ethiopia; 3 OIE Reference Laboratory for Contagious Bovine Pleuropneumonia, Instituto Zooprofilattico Sperimentale of Abruzzo and Molise, via Campo Boario, 64100 Teramo, Italy; 4 International Livestock Research Institute (ILRI), P.O. Box 5689, Addis Ababa, Ethiopia

**Keywords:** CCPP, cELISA, Risk factors, Sero-prevalence

## Abstract

From November 2016 to April 2017, a cross-sectional study to determine the sero-prevalence of contagious caprine pleuropneumonia (CCPP) and to investigate its epidemiology was conducted in selected districts of Borana zone in Ethiopia. In addition, the study aimed at identifying *Mccp* antigens using species specific primer of PCR. A multistage random sampling was implemented to select districts, pastoral associations (villages), and households. A total of 890 serum samples of small ruminants that had not been vaccinated (goats *n* = 789 and sheep *n* = 101) were collected and screened for the presence of antibodies against *Mycoplasma capricolum* subspecies *capripneumoniae* using a competitive enzyme-linked immunosorbent assay. Lung tissues and pleural fluid samples were collected from 3 sero-positive and clinically suspected goats for isolation of *Mycoplasma capricolum* subspecies *capripneumoniae*. Serology showed that overall 31.2% (246/789) of goats and 12.9% (13/101) of sheep were positive with statistically significant differences between districts (*p* = 0.001). Multivariable logistic regression analysis revealed that goats from Moyale and Yabello districts had higher odds of being positive than goats from Elwoya district with odd ratios of 2.05 and 1.61, respectively. Age of goats was also significantly associated with sero-positivity (OR = 1.47; CI 95% 1.2–1.8). *Mycoplasma capricolum* subspecies *capripneumoniae* was identified in 6 (75%) of the tissue samples using species-specific primer of PCR. Besides improving the understanding of the epidemiology of CCPP in the selected districts and demonstrating its wide distribution, the study highly also provides evidence of the possible role of sheep in the maintenance of the disease.

## Introduction

Contagious caprine pleuropneumonia (CCPP) caused by *Mycoplasma capricolum* subspecies *capripneumoniae* (*Mccp*) is a severe and devastating respiratory disease with high morbidity and mortality in goats (Sadique et al. 2012; Tsehay et al. 2014), causing considerable economic losses (Asmare et al. 2016). It occurs in many countries in Africa, Asia, and Middle East (Prats-van der Ham et al. 2015) and is a classical trans-boundary animal disease (Shahzad et al. 2016). Moreover, the disease is included in the list of notifiable diseases of the World Organization for Animal Health (OIE 2008) as it threatens a significant number of goat populations throughout the world and has a considerable socioeconomic impact in infected territories (Atim et al. 2016). Though disease is mainly found in goats, subclinical cases were reported in sheep and some wild ruminant species (Asmare et al. 2016).

The classical disease caused by *Mycoplasma capricolum* subspecies *capripneumoniae* (*Mccp*) is predominantly respiratory (Thiaucourt et al. 1996). Typical cases of CCPP are characterized by extreme fever (41–43 °C), and high morbidity and mortality in susceptible herds affecting all ages (AU-IBAR 2015). Associated common clinical signs are anorexia, weakness, emaciation, dullness, exercise intolerance, and respiratory signs such as dyspnea, polypnea, coughing, and nasal discharges (Shahzad et al. 2016). Further, abortion and high mortality rates have been reported (Wazir et al. 2016).

Commonly used serological tests are indirect hemagglutination, complement fixation, and latex agglutination (LAT) to detect the antibody response of goats to Mccp (Samiullah 2013). Recently, a competitive enzyme-linked immunoassay (cELISA) for CCPP has been developed and found highly specific (Peyraud et al. 2014). The introduction of the cELISA for CCPP will permit the implementation of serological studies on a large scale (Younis et al. 2015). In addition to serological tests, molecular detection of *Mccp* directly in clinical samples was found highly sensitive and specific and should be used for diagnosis of CCPP, especially in outbreaks to confirm the disease for rapid control (Elhassan and Salama 2018).

In Ethiopia, goats play a unique role in the livelihood of pastoral communities, especially for women, as they provide milk and dairy products and are a source of income for the family to cover school fees for children and other family expenses. Despite the presence of a massive goat population and their important socio-economic role, health of small ruminants in general and goats in particular has received little attention so far (Lakew et al. 2014). Only few studies have been carried out in the area, but these showed that CCPP is prevalent and causes considerable mortality in goats. For instance, between 2011 and 2015, 83 outbreaks affecting 23,950 goats were reported (MoLF 2016). Hence, reliable epidemiological information is needed in order to design effective control measures. Specifically, antigen detection of *Mccp* and the role of sheep in the maintenance of the disease need to be explored. The objectives of the study were to assess the epidemiology of CCPP in the Borana zone and to characterize the causative agent using molecular techniques.

## Materials and methods

This study was conducted in the Borana zone that is predominantly inhabited by the Borana community and extends to the Kenyan border in the South; Somali region in the South East; Southern Nation, Nationalities, and People Region (SNNPR) in the West and North; and Guji zone in the North East. Borana rangeland is characterized by a semiarid to arid climate (Kamara et al. 2005; Haile et al. 2011). Geographically, the area is located between from 4 to 6° N latitude and from 36 to 42° E longitude with altitude ranging from 1000 to 1700 m above sea level. The mean annual rainfall of the area ranges from 250 to 700 mm. The annual mean temperature varies from 19 to over 25 °C. Extensive pastoralism is the main means of livelihoods for the Borana people (Gelagay et al. 2007).

**Fig. 1:**
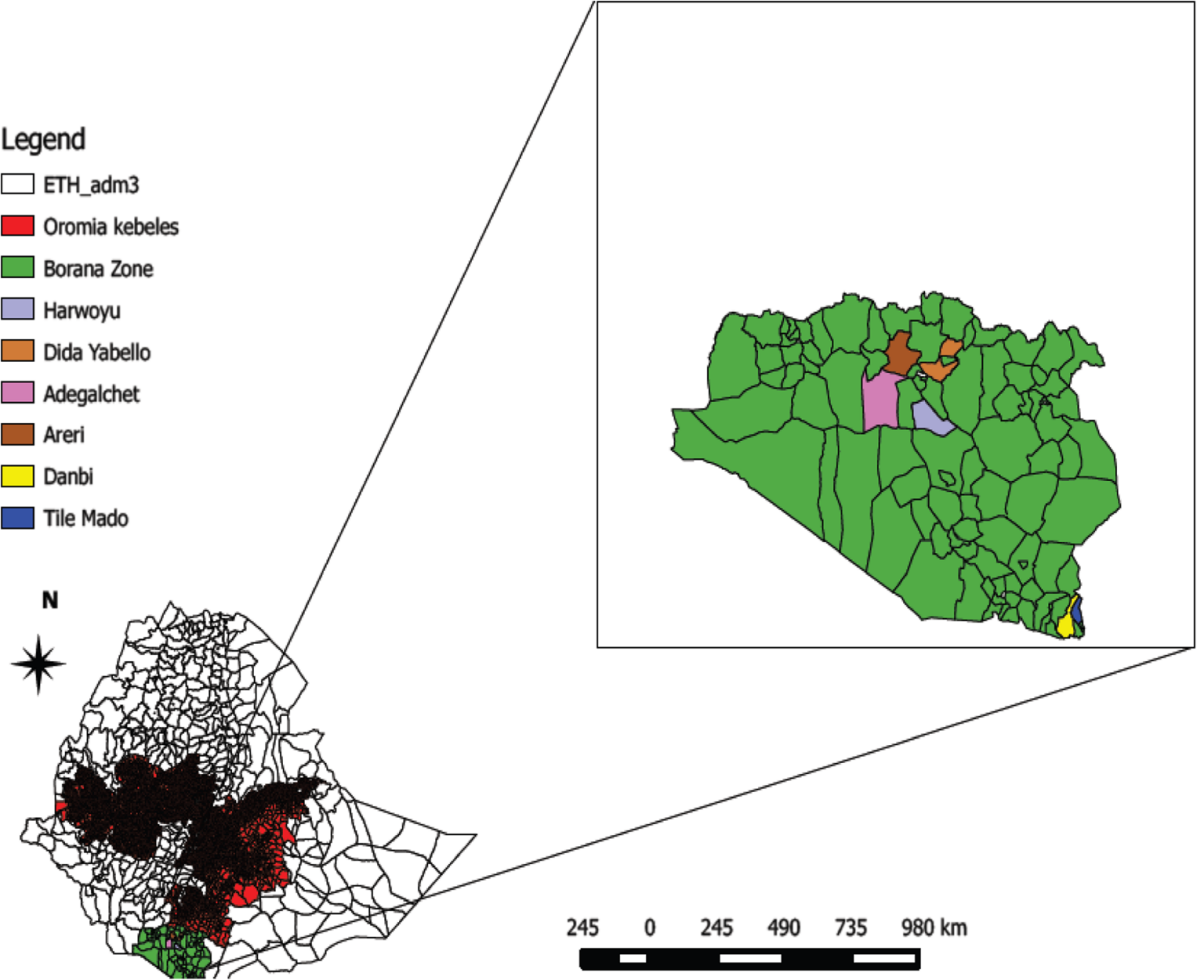
Map of Ethiopia showing study areas

Multistage random sampling was applied to select the study animals. The sampling frame comprised a list of all districts in the zone and pastoral associations (PAs) or villages. Three districts were selected randomly, and in each of them, two PAs where no CCPP vaccination had been conducted for more than 2 years were selected. The resulting six PAs/villages were Areri and Adegalchet from Elwoya, Tile Mado and Dambi from Moyale, and Dida Yabello and Harwoyu from Yabello (Fig. 1).

Finally, data were collected from a total of 161 households residing in the study villages. The distribution of households across the villages was 29, 30, 29, 20, 30, and 23 households from Adegalchet, Areri, Dambi, Tile Mado, Dida Yabello, and Harwoyu respectively. A total of 789 goats from 161 households in the selected PAs were sampled. Beside serum sample collection from the districts, randomly selected households (*n* = 161) who have small ruminants were interviewed using semi-structured questionnaire to capture general information they had on CCPP. If the flock size of a household was greater than five, 4 to 9 goats were selected from each flock whereas all goats per household were sampled if the flock size was less than or equal to five. In addition, 101 in contact sheep were selected purposively. The goats selected were identified with ear tags and information on household profiles and attributes of animals was collected before sampling. The age of animals was estimated using information from owner and dentition. Besides sero-samples, pleural fluids, and lung tissue samples were collected from sero-positive, clinically affected goats for molecular and bacteriological investigations. During sampling, recently introduced animals were excluded to avoid the risk of including vaccinated animals. To categorize flock size into small, medium, and large, five key informants were used from each district.

### Sample size estimation

The estimation of sample size for epidemiological investigation using serological assay was done using the formula given by Thrusfield (2005) considering 95% confidence level, expected prevalence of 31.6% (Lakew et al. 2014) and 5% absolute precision.

$$ n=\frac{1.96^2{P}_{\mathrm{exp}}\;\left(1-{P}_{\mathrm{exp}}\right)}{d^2} $$

where,

*n*: required sample size
*P*_exp_: expected prevalence
*d*: desired absolute precision (5%)

Accordingly, a minimum of 332 goats was obtained. To account for intra-class correlation at herd, village, and district levels, a design effect of 2 was considered, resulting in a minimum sample size of 664 (calculated with EpiInfo 7.2).

### Blood sample collection

Approximately 5–7 mL of blood was collected from jugular vein of apparently healthy goats and sheep not involved in vaccination against CCPP for at least 2 years for serological examination using sterile vacutainer tubes and needles. Samples were then transported in an icebox to the microbiology laboratory of the Yabello Pastoral and Dryland Agriculture Research Center. The sera were separated after centrifugation at 1500 rpm for 10 min. The serum samples were collected into sterile cryogenic tubes and stored at − 20 °C until the samples were transported to the National Animal Health Diagnostic and Investigation Center (NAHDIC), Sebeta, Ethiopia, for analysis.

### Collection of tissue samples

Three goats that were positive in the cELISA test or which were suspected to be clinically affected by CCPP after thorough clinical examination were purchased and sacrificed for postmortem examination. Gross pathological lesions were observed and samples of lung at the interface between the consolidated and unconsolidated healthy tissues and pleural fluids were collected and transported to the National Veterinary Institute (NVI), Bishoftu, Ethiopia, for molecular analysis using polymerase chain reaction (PCR) as described by Woubit et al. (2004).

### Laboratory analysis of samples

The serum samples were examined for the presence of specific antibodies against *Mccp* by using a commercial cELISA (Idexx, France), according to the instructions of the manufacturer. The test is characterized by a specificity of 99.9%. At the end of the reactions, ELISA plates were read at 450 nm by BioTek ELx800 ELISA reader to determine the optical density and percentage of inhibition was calculated. Samples with percentage of inhibition greater than or equal to 55% were considered positive (Peyraud et al. 2014).

### Polymerase chain reaction

Samples for polymerase chain reaction (PCR) were prepared as described by Woubit et al. (2004). About 1 g samples from each lung tissue and bronchial lymph nodes was taken and chopped with scissors and then grinded by mortar and pestle; mixed with 9 mL phosphate buffer solutions (PBS) and transferred to test tubes. For pleural fluids, 1 mL of pleural fluid was taken and mixed with 9 mL PBS and subjected for DNA extraction. Primers used (Mccp-spe-F, 5′-ATCATTTTTAATCCCTTCAAG-3′ and Mccp-spe-R, 5′-TACTATGAGTAATTATAATA-TATGCAA-3′) amplify a DNA fragment of 316 bp; PCR conditions were set as described by Woubit et al. (2004).

### Data analysis

Data collected from the field and laboratory assays were entered and stored in Microsoft Excel spreadsheet, screened for proper coding and errors, and analysis was done. Disease prevalence and odds ratio were calculated using STATA 13.0 (Stata Corp. 1985–2013) statistical software. Logistic regression analysis was used to measure association between potential risk factors and sero-prevalence. Variables with *p* value of less than 0.05 were included in multivariable analysis and multivariable model was fitted. Finally, odd ratios and 95% confidence interval were calculated and disease-associated risk factors with a *p* value less than 0.05 considered significant.

## Results

### Survey result on symptoms of CCPP observed

**Fig. 2:**
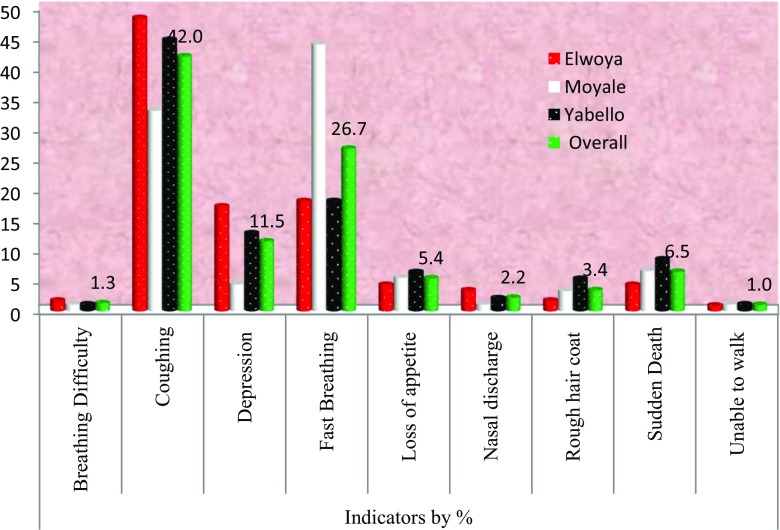
Clinical symptoms of CCPP as reported by respondents (*N* = 161)

During the current survey, different and common overall symptoms of CCPP mentioned by respondents in the three study districts are coughing, fast breathing, depression, sudden death, inappetance, diarrhea, rough hair coat, nasal discharge difficulty in breathing, and reluctant to walk with 42%, 26.7%, 11.5%, 6.5%, 5.4%, 3.4%, 2.2%, 1.3%, and 1% respectively as indicated in Fig. 2.

### Sero-prevalence and associated risk factors for CCPP in goats

**Fig. 3:**
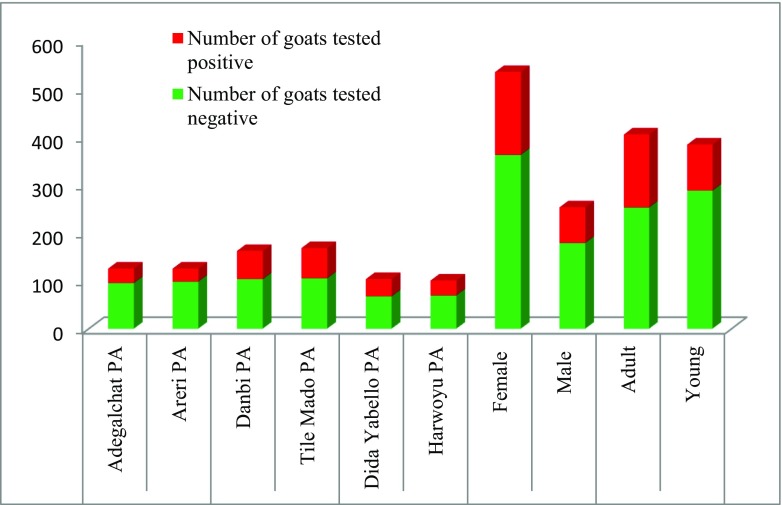
Proportion of sero-positivity in goats by locality

From the 161 households involved in the study, sera of 789 animals were collected. Sero-positivity was detected in all localities surveyed as depicted in Fig. 3. Two hundred forty-six (31.2%) of collected sera tested positive for anti-*Mccp* antibodies. The highest prevalence (36.70%) was observed in Moyale district, followed by Yabello (32.7%) and Elwoya (22.6%) (Table 1). The difference in sero-prevalence between districts was statistically significant (*p* = 0.001). There was also a significant difference in the sero-prevalence CCPP between different age groups (*p* < 0.001) in which adult goats (37.3%) were more likely to test positive than young goats (24.7%). Higher sero-prevalence was recorded in female goats (32.1%) than in males (29.1%) although this difference was not statistically significant. Similarly, sero-prevalence of CCPP was 34.3%, 32.2%, and 28.8% in small, medium, and large flock sizes, respectively. However, the difference in prevalence among various flock sizes was not statistically significant.

**Table 1:** Results of univariable analysis to identify risk factors of sero-prevalence of CCPP in goats in Borana zone, Oromia, Ethiopia

Risk factors	Number	Test positive	Prevalence	*X* ^2^	*p* value
District				13.618	0.001
Elwoya	252	57	22.6		
Moyale	332	122	36.7		
Yabello	205	67	32.7		
Sex				0.73	0.393
Female	535	172	32.1		
Male	254	74	29.1		
Age				14.455	< 0.001
Adult	405	151	37.3		
Young	384	95	24.7		
Flock size					
Small	175	60	34.3	1.82	0.402
Medium	267	86	32.2		
Large	347	100	28.8		
Overall	789	246	31.2		

Fitting a multivariable regression model revealed that among the risk factors considered in the analysis (Table 2), district and age were associated with sero-positivity (*p* < 0.05), whereas sex and flock size had no statistically significant effect. The results showed that animals in Moyale and Yabello districts had about twice and 1.6 times higher odds of being positive for CCPP, respectively, than those animals reared in Elwoya district. Similarly, the odds of CCPP sero-prevalence was observed to significantly increase by 1.5 times as age of animals increase by 1 year (Table 2).

**Table 2:** Results of multivariate logistic regression analysis of sero-prevalence of CCPP in goats

Risk factor	Odds ratio	Std. Err.	*z*	*p* > |*z*|	(95% confidence interval)
District					
Moyale	2.050	0.3982	3.7	< 0.001	(1.401–2.999)
Yabello	1.611	0.3457	2.22	0.026	(1.058–2.453)
Sex					
Male	0.924	0.157	− 0.47	0.64	(0.662–1.289)
Age in year	1.472	0.157	3.64	< 0.001	(1.195–1.814)
Flock size					
Medium	1.172	0.211	0.88	0.378	(0.823–1.669)
Small	1.429	0.297	1.72	0.086	(0.951–2.146)
_cons	0.137	0.036	− 7.55	0.000	(0.081–0.229)

### Sero-prevalence and associated risk factors of CCPP in sheep

From a total of 101 serum samples collected from apparently healthy sheep and tested by cELISA, 13 (12.9%) were found positive. The differences in sero-prevalence between age groups, sex, and districts examined were not statistically significant (*p* > 0.05) as presented in Table 3.

**Table 3:** Results of multivariable logistic regression analysis of associated risk factors of CCPP in sheep in the study area

Risk factors	Odds ratio	Std. Err.	*z*	*p* > |*z*|	(95% Conf. interval)
Age in year	1.185	0.603	0.33	0.738	(0.437–3.213)
Sex					
Male	0.320	0.274	− 1.33	0.184	(0.059–1.715)
District					
Moyale	1.015	0.751	0.02	0.984	(0.238–4.329)
Yabello	0.885	0.697	− 0.15	0.877	(0.189–4.138)
_cons	0.150	0.163	− 1.74	0.081	(0.018–1.264)

### Results of gross pathological examination

**Fig. 4:**
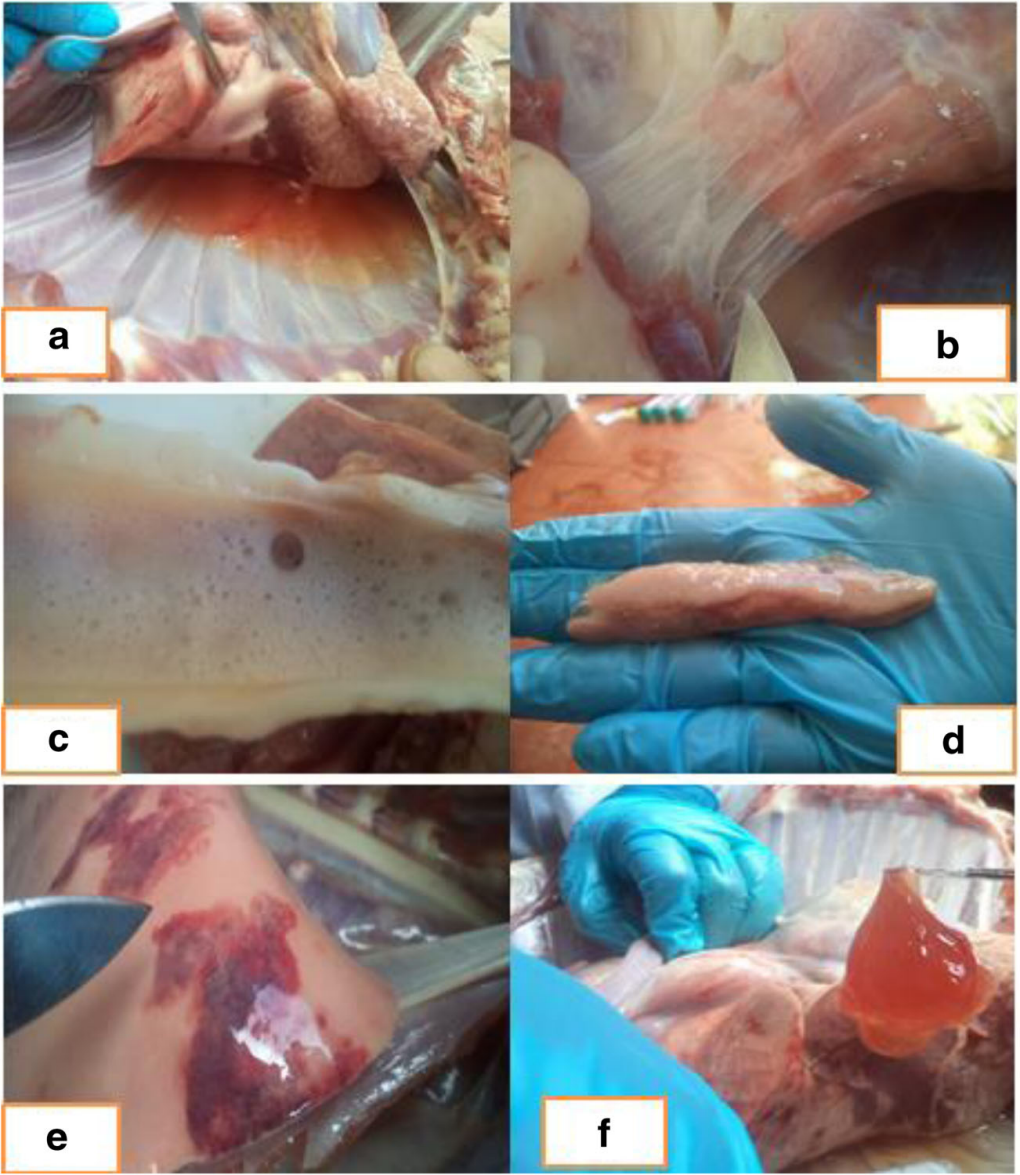
Postmortem finding of CCPP infected goats. **a** Accumulation of lung exudate in thorax cavity; **b** fibrous adhesion of lungs to the chest wall; **c** froth in the trachea; **d** enlarged respiratory (mediastinal) lymph nodes; **e** lung with areas of pneumonia; and **f** lung exudate containing large clots of fibrin

Gross pathological changes observed in three goats showing clinical signs of CCPP include accumulation of fluids in the pleural cavities, adhesion of lungs to the thoracic wall, frothy discharge in the trachea, enlarged bronchial lymph nodes, pneumonic lung tissues, and pleural fluids containing large clots of fibrin (Fig. 4).

### Mccp detection and confirmation using conventional PCR

A total of 8 samples (three lung tissues, three pleural fluids, and two bronchial lymph nodes) collected from three clinically affected goats that tested positive in the cELISA were analyzed by conventional PCR. Upon PCR amplification of the genomic DNA from the 8 samples and controls using species-specific Mccp primers, Mccp was detected in 6 (75%) samples.

**Fig. 5:**
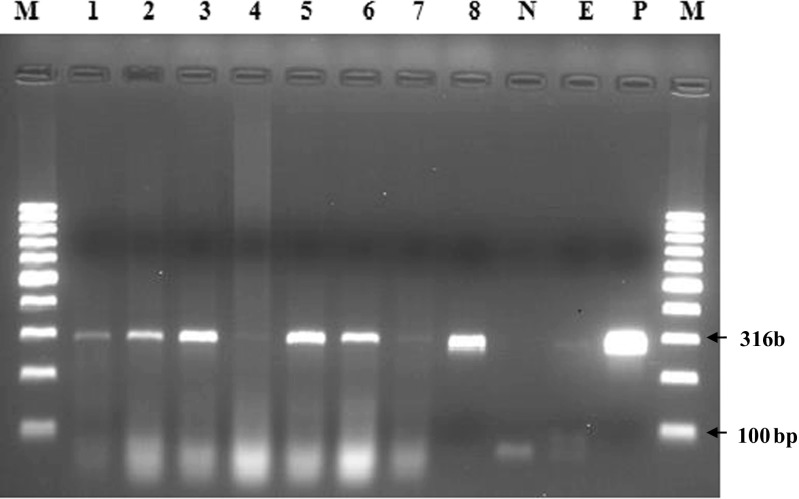
Agarose gel electrophoresis of PCR products (316 bp) amplified with *Mccp*-specific primers. Lane M: 100 bp DNA molecular weight marker; lane P: positive control; lane N: negative control; lane E: extraction control; lanes 1–8: samples

The specimens that tested positive include three lung tissues (lane 1–3) and three pleural fluids (lane 5, 6, and 8) whereas the samples of the other bronchial lymph node (lane 4 and lane 7) tested negative. The results of PCR analysis are depicted in Fig. 5. The fragment size of the amplified products was 316 bp.

## Discussion

The main objective of this study was to estimate the sero-prevalence and confirm the presence of CCPP in selected districts of Borana zone. The study revealed that CCPP is a major health constraint of goats in Borana pastoral areas. This was confirmed by a general sero-prevalence of 31.2% and by the detection of *Mccp* in the samples collected from the three suspected cases. The current study indicated the confirmation of the case directly from clinically affected goats for the first time in the study area. It has been shown previously that several outbreaks of CCPP reported in the country were from Oromia, the majority of which were from Borana (MoLF 2016). The previous reports of outbreaks were based on the clinical signs. This study, however, provided confirmation of CCPP cases with molecular techniques and provided reliable information on the presence of *Mccp* in Borana area. This has important implication for the wellbeing of the pastoral community.

The overall sero-prevalence of 31.2% reported in unvaccinated goats in this study shows that *Mccp* has been established and is circulating in the area. For unvaccinated population of goats this figure is high and requires attention of the veterinary and livestock authority of the area to minimize the effect CCPP has on livelihoods in the community. The overall prevalence of CCPP in the present study was higher than the national prevalence estimated from pooled sero-prevalence (25.7%) through a systematic review by Asmare et al. (2016) and is largely in agreement with the previous findings from Ethiopia (Lakew et al. 2014) in which 31.6% of goats in Borana were found to be positive to CCPP. Similar observations were also made earlier in goats at an export abattoir at Bishoftu, Ethiopia (Eshetu et al. 2007), and Southern Ethiopia, in Tigray and Afar (Hadush et al. 2009), and in Beetal goats in Pakistan (Sherif et al. 2012; Hussain et al. 2012). Thus, our findings show that little has changed over the years, and the efforts made to control the disease with vaccinations have not resulted in sufficient vaccination coverage to prevent spread or contain the disease. This was also reflected by the fact, that it was easy to find villages in which goats had not been vaccinated against CCPP.

In contrast to our findings, lower prevalence of CCPP has been reported earlier from different parts of Ethiopia (Yousuf et al. 2012; Tesfaye et al. 2011; Mekuria et al. 2008; Mekuria and Asmare 2010; Aklilu et al. 2015; Regassa et al. 2010). Lower CCPP sero-prevalence has also recently described in Pakistan (Shahzad et al. 2016; Wazir et al. 2016). On the other hand, higher sero-prevalence of 44.5%, 47.3%, and 51.8% was reported from Dire Dawa, Afar, and Oromia regions of Ethiopia, respectively, by Gizawu et al. (2009). Hadush et al. (2009) also reported higher prevalence as 38.6% and 43.9% from Afar and Tigray regions of Ethiopia, respectively. In other parts of the world, higher prevalence than our observation has been documented in Beetal, Pakistan (Shahzad et al. 2012), Tanzania (Mbyuzi et al. 2014; Nyanja et al. 2013), Kenya (Kipronoh et al. 2016), Uganda (Atim et al. 2016), and Turkey (Cetinkaya et al. 2009). An international collaborative study done by Peyraud et al. (2014) also reported sero-prevalence of 6 to 90%, 14.6%, 16%, 10.1%, 0%, and (2.7%, 44.2%) from Kenya, Ethiopia, Mauritius, Tajikistan, Afghanistan, and Pakistan, respectively, using monoclonal antibody–based cELISA. The observed variation in sero-prevalence reported from different studies may be due to differences in the husbandry practices, agro-ecology, vaccination history, sampling methods applied, and sample size used.

In our study, the sero-prevalence of CCPP was significantly lower in Elwoya than in Moyale and Yabello. This observation agrees with the reports of Wazir et al. (2016) who reported significantly different prevalence among geographical areas. However, it is contrary to the previous findings (Eshetu et al. 2007; Hadush et al. 2009; Sherif et al. 2012). The higher prevalence in Moyale and Yabello compared to Elwoya observed in this study could be due to differences in frequency of animal movement in the districts. Moyale is a district bordering Kenya. There is free movement of animals between the two countries in search of market and pastures. Pastoralists in the area often cross the border for marketing purposes as well as in search of feed and water mostly during the dry season and during droughts. There is also free movement and contact with animals from neighboring Somali pastoralists in Moyale. Yabello is the center of Borana zone, where animals from surrounding PAs are being moved to for veterinary services and marketing. Therefore, the higher prevalence of CCPP in these two districts is probably due to animal movement for marketing and in search of water and pasture.

The serological test results showed the presence of anti-*Mccp* antibodies in all age groups of goats and sheep. However, the results of sero-prevalence study showed that age had significant effect on the occurrence of infection with *Mccp* in Borana zone, reflecting the fact that older animals have higher chances to be exposed to the pathogen. This observation is in consent with the findings of Aklilu et al. (2015) who reported that adult goats were 1.84 times more likely to be sero-positive than kids. Our findings also agree with the report of Mekuria and Asmare (2010), Bekele et al. (2011), Yousuf et al. (2012), Sherif et al. (2012), Nyanja et al. (2013), and Lakew et al. (2014) who observed the presence of significant variation among age groups. However, the finding of this study contradicts with the works of Gizawu et al. (2009), Nicholas (2002), Eshetu et al. (2007), and Hadush et al. (2009) who observed the absence of association between age and occurrence of CCPP.

In this study, sheep kept along with goats were found to be sero-positive in all PAs except in Areri. That is, sheep in contact with infected goats were sero-positive. In consent with our observation, previous authors showed that sheep were sero-positive from different parts of Ethiopia. For instance, 13% of sheep were found sero-positive by Dawit (1996), 7.14% by Gelagay et al. (2007), and 47.6% by Hadush et al. (2009). In Tanzania, sero-prevalence estimates of 36.7% and 22.9% from sheep serum were reported by Mbyuzi et al. (2014). In addition to this, there are reports describing the isolation of *Mcpp* from sheep with respiratory disease returning to Eritrea with refugees from Sudan (Houshaymi et al. 2002), from healthy sheep in Kenya that have been in contact with goat herds affected by CCPP (Litamoi et al. 1990), from sick sheep mixed with goats in Uganda (Bolske et al. 1995), and elsewhere in the globe by Cetinkaya et al. (2009) from lung and nasal swab of sheep. This raises questions on the role of sheep as a reservoir and contributing to maintaining transmission of *Mccp*. The exact role of sheep in the maintenance and spread of *Mccp* to goats needs to be further investigated.

Our finding of CCPP gross lesions at postmortem which revealed lung exudate containing large clots of fibrin, adhesion of lungs to the thoracic wall, froth in the trachea, enlarged bronchial lymph nodes, and pneumonic lung tissues are similar with those of the previous study of Wesonga et al. (2004) who reported the lesions of classical CCPP caused by *Mccp*. These observations are also matched with the findings of others (OIE 2008; Sadique et al. 2012).

In conclusion, the present study revealed the prevalence of CCPP in the Borana pastoral area.

The causative agent of CCPP, *Mycoplasma capricolum* subspecies *capripneumoniae*, was identified and confirmed by PCR*.* The study also showed that sheep were infected with *Mccp* with a sero-prevalence of 12.9%. Based on our and previous studies, it is clear that CCPP represent a priority for goat farming and more coordinated efforts are needed to prevent the disease and mitigate its impact. In addition, further studies on economic impact of the disease on production performance of goats and studies focused on molecular characterization of the circulating strain for both sheep and goats using large sample size should be done.
